# Supplementary material for: Adaptive genetic variation at three loci in South African vervet monkeys (Chlorocebus pygerythrus) and the role of selection within primates
Source: PeerJ. 2018 Jun 4;6:e4953. doi: 10.7717/peerj.4953 (PMC5991302; doi:10.7717/peerj.4953)
Supplement: Supplemental Information 1 [file peerj-06-4953-s001.docx]

**Title: Adaptive genetic variation at three loci in South African vervet monkeys (Chlorocebus pygerythrus) and the role of selection within Primates**

**Authors:** Willem G. Coetzer, Trudy R. Turner, Christopher A. Schmitt and J. Paul Grobler

**Supplementary data.** Haplotype DNA sequences for vervet *ACR*, *TLR4* and *TLR7* generated during the current study.

>ACR_Hap_1

GGGCGCATTCAGCCAACCAATGTGTGCGCGGGGTATCCTTTAGGCAAGATCGACACCTGCCAGGTAACCTTCCTTCTGGCTTCTGGGCCCCTGGGTCCCTCCGGGACTCTCCCGGCCCCTGAGAACATCCTCATTTTGGATCCCCAAGCTCCATTATCTCCACCCCTCTGCCTAGGGCCCTTCTCTAGTGACTGCTTCCCTGGTCCCTTTTCTAGCACCTACAGTCACAGTGGGGATGAGAGGTGGCAGCCACGGGCAGCCCCTGCCATGTGCCCCTATGGACACGTGCGCTTGCTCATCTCACTGCAAGGAAAGCCCTGACAATCCACACCCTCCTCACATCCCAAATGAAGCCCTTGACACCCCCTCAAACTTTACCACAACCACTTGTGTTTATGGCAGCAGGAAACCATGTGACTGTGGAAATTGTCCTCCCAGAGCCTTCTGACCCCTCTTGGCAGGGAAAGAGTGGTTCAGGCAGGTCGTGACCTCTGTGTCCTTCTGGGCAGGGGGACAGTGGCGGGCCTCTCATGTGCAAAGACAGCAAGAAAGGCGCCTACGTGGTCGTGGGAATCACAAGCTGGGGGGTAGGCTGTGCCCGTGCCAAGCGCCCCGGAATCTACACGGCCACCTGGCCCTATCTGAACTGGATTGCCTCCAAGATTGGTTCTAATGCTCTGCATATGATTCAACCGGCCACCCCTCCACCCCCCACCACTAGACCGCCCCCAATTCGACTGCCCTCCTCCCACCCTATCTCTGCTCACCTTCCTTGGTATTTCCAACCGCCCCCTCGACCACTTCCATCCCGACCACCTTCAGCTCAGCCTCGACCCCCACCTCCACCCTCACCTTTACCTCCACCCCCGCCCCCACCTCCACCCTCATCTACCACAAAACCTCCCCAAGGACTTTCTTTTGCCAAGCGCCTACAGCAGCTCATAGAGGCCTTGAAGGGGAAGACCTATTCCGACGGAAAGAACTATTATGATATGGAGACCACAGAGCTCCCAGAACTGACCTCTACCTCCTGA

>ACR_Hap_2

GGGCGCATTCAGCCAACCAATGTGTGCGCGGGGTATCCTTTAGGCAAGATCGACACCTGCCAGGTAACCTTCCTTCTGGCTTCTGGGCCCCTGGGTCCCTCCGGGACTCTCCCGGCCCCTGAGAACATCCTCATTTTGGATCCCCAAGCTCCATTATCTCCACCCCTCTGCCTAGGGCCCTTCTCTAGTGACTGCTTCCCTGGTCCCTTTTCTAGCACCTACAGTCACAGTGGGGATGAGAGGTGGCAGCCACGGGCAGCCCCTGCCATGTGCCCCTATGGACACGTGCGCTTGCTCATCTCACTGCAAGGAAAGCCCTGACAATCCACACCCTCCTCACATCCCAAATGAAGCCCTTGACACCCCCTCAAACTGTACCACAACCACTTGTGTTTATGGCAGCAGGAAACCATGTGACTGTGGAAATTGTCCTCCCAGAGCCTTCTGACCCCTCTTGGCAGGGAAAGAGTGGTTCAGGCAGGTCGTGACCTCTGTGTCCTTCTGGGCAGGGGGACAGTGGCGGGCCTCTCATGTGCAAAGACAGCAAGAAAGGCGCCTACGTGGTCGTGGGAATCACAAGCTGGGGGGTAGGCTGTGCCCGTGCCAAGCGCCCCGGAATCTACACGGCCACCTGGCCCTATCTGAACTGGATTGCCTCCAAGATTGGTTCTAATGCTCTGCATATGATTCAACCGGCCACCCCTCCACCCCCCACCACTAGACCGCCCCCAATTCGACTGCCCTCCTCCCACCCTATCTCTGCTCACCTTCCTTGGTATTTCCAACCGCCCCCTCGACCACTTCCATCCCGACCACCTTCAGCTCAGCCTCGACCCCCACCTCCACCCTCACCTTTACCTCCACCCCCGCCCCCACCTCCACCCTCATCTACCACAAAACCTCCCCAAGGACTTTCTTTTGCCAAGCGCCTACAGCAGCTCATAGAGGCCTTGAAGGGGAAGACCTATTCCGACGGAAAGAACTATTATGATATGGAGACCACAGAGCTCCCAGAACTGACCTCTACCTCCTGA

>TLR4_Hap_1

AACTTTATCCAACCAGGTGCATTTAAAGAAATTAGGCTTCATAAGCTGACTTTGAGAAATAATTTTGATGATTTAAATGTGATGAAAACTTGTATTCAAGGTCTGGCTGGTTTAGAAGTCCATCGTTTGGTTCTGGGAGAATTTAGAAATGAAAGAAACTTGGAAGAGTTTGACAAATCTGCTCTGGAGGGATTGTGCAATTTGACCATTGAAGAATTCCGATTAACATACTTAGACTACTACCTCAATAATATTATTGACTTATTTAATTGTTTGGCAAATGTTTCTTCATTTTCCCTGGTGAGTGTGAATATTAAAAGGGTAGAAGACTTTTCTTATAATTTCAGATGGCAACATTTAGAATTAGTTAAGTGTAAATTTGAACAGTTTCCCACATTGGAACTCAAATCTCTCAAAAGGCTTACTTTCACTGCCAACAAAGGTGGGAATGCTTTTTCAGAAGTTAATCTACCAAGCCTTGAGTTTCTAGATCTCAGTAGAAATGGCTTGAGTTTCAAAGGTTGCTGTTCTCAAAATGATTTTGGGACAACCAGCCTAAAGTATTTAGATCTGAGCTTCAATGAT

>TLR4_Hap_2

AACTTTATCCAACCAGGTGCATTTAAAGAAATTAGGCTTCATAAGCTGACTTTGAGAAATAATTTTGATGATTTAAATGTGATGAAAACTTGTATTCAAGGTCTGGCTGGTTTAGAAGTCCATCGTTTGGTTCTGGGAGAATTTAGAAATGAAAGAAACTTGGAAGAGTTTGACAAATCTGCTCTGGAGGGATTGTGCAATTTGACCATTGAAGAATTCCGATTAACATACTTAGACTACTACCTCAATAATATTATTGACTTATTTAATTGTTTGGCAAATGTTTCTTCATTTTCCCTGGTGAGTGTGAATATTAAAAGGGTAGAAGACTTTTCTTATAATTTCAGATGGCAACATTTAGAATTAGTTAAGTGTAAATTTGAACAGTTTCCCACATTGGAACTCAAATCTCTCAAAAGGCTTACTTTCACCGCCAACAAAGGTGGGAATGCTTTTTCAGAAGTTAATCTACCAAGCCTTGAGTTTCTAGATCTCAGTAGAAATGGCTTGAGTTTCAAAGGTTGCTGTTCTCAAAATGATTTTGGGACAACCAGCCTAAAGTATTTAGATCTGAGCTTCAATGAT

>TLR4_Hap_3

AACTTTATCCAACCAGGTGCATTTAAAGAAATTAGGCTTCATAAGCTGACTTTGAGAAATAATTTTGATGATTTAAATGTGATGAAAACTTGTATTCAAGGTCTGGCTGGTTTAGAAGTCCATCGTTTGGTTCTGGGAGAATTTAAAAATGAAAGAAACTTGGAAGAGTTTGACAAATCTGCTCTGGAGGGATTGTGCAATTTGACCATTGAAGAATTCCGATTAACATACTTAGACTACTACCTCAATAATATTATTGACTTATTTAATTGTTTGGCAAATGTTTCTTCATTTTCCCTGGTGAGTGTGAATATTAAAAGGGTAGAAGACTTTTCTTATAATTTCAGATGGCAACATTTAGAATTAGTTAAGTGTAAATTTGAACAGTTTCCCACATTGGAACTCAAATCTCTCAAAAGGCTTACTTTCACTGCCAACAAAGGTGGGAATGCTTTTTCAGAAGTTAATCTACCAAGCCTTGAGTTTCTAGATCTCAGTAGAAATGGCTTGAGTTTCAAAGGTTGCTGTTCTCAAAATGATTTTGGGACAACCAGCCTAAAGTATTTAGATCTGAGCTTCAATGAT

>Hap_1

CCAGCGTCCTTTCACAGACTGGTCCATCTGGTAGAGATCGATTTCAGATGCAACTGTGTACCTATTCGATTGGGGTCAAAAAGCAACATGTGCCCCAGGAGGCTGCAGATTAAACCCAGAAGCTTTAGTGGACTCACTTATTTAAAATCCCTTTACCTGGATGGAAACCAGCTTCTAGAGATACCGCAGGGCCTTCCACCCAGCTTACAGCTTCTCAGCCTTGAGGCCAACAACATCTTTTCCATCAGAAAAGAGAATCTAACAGAACTGGCCAACATAGAAATACTCTATCTGGGCCAAAACTGTTATTATCGAAATCCTTGTTATGTTTCATATTCAATAGAAAAAGATGCCTTCCTAAACTTGACAAAGTTAAAAGTGCTCTCCCTGAAAGATAACAATGTCACAACCGTCCCTACTGTTTTGCCATCTACTTTAACAGAACTATATCTCTACAACAACATGATTGCAGAAATCCAAGAAGATGATTTTAATAACCTCAACCAATTACAAATTCTTGACCTAAGTGGAAATTGCCCTCGTTGTTATAATGCCCCATTTCCTTGTACGCCATGTAAAAATAATTCTCCCCTACAGATCCCTGTAAATGCTTTTGATGCGCTGACAGAATTAAAAGTTTTACGTCTACACAGTAACTCTCTTCAGCATGTGCCCCCAAGATGGTTTAAGAACATCAACAATCTCCAGGAACTAGATCTGTCCCAAAACTTCTTGGCCAAAGAAATTGGGGATGCCAAATTTCTGCATTTTCTCCCCAACCTCATCCAATTGGATCTGTCTTTCAATTTTGAACTTCAGGTCTATCGTGCATCTATGAATCTATCACAAGCATTTTCTTCACTGAAAAGCCTGAAAATTCTGCGGATCAGAGGATATGTCTTCAAAGAGCTGAAAAGCTTTAACCTCTCTCCATTACACAATCTTCAAAATCTTGAAGTTCTTGATCTTGGTACTAACTTTATAAAAATTGCTAACCTCAGCATGTTTAAACAATTTAAAAGATTGAAAGTCATAGATCTTTCAGTGAATAAAATATCACCTTCAGGAGATTCAAGTGAAGTTGGCTTCTGCTCAAATGCCAGAACTTCTGTAGAAAGTTATGAACCCCAGGTCCTGGAACAATTATATTATTTCAGATATGATAAGTATGCAAGGAGTTGCAGGTTCAAAAACAAAGAGGCTTCTTTCACGTCTGTTAATGAAAGCTGCTACAAGTATGGGCAGACCTTGGATCTAAGTAAAAATAGTATATTTTTTATCAAGTCCTCTGATTTT

>Hap_2

CCAGCGTCCTTTCACAGACTGGTCCATCTGGTAGAGATCGATTTCAGATGCAACTGTGTACCTATTCGATTGGGGTCAAAAAGCAACATGTGCCCCAGGAGGCTGCAGATTAAACCCAGAAGCTTTAGTGGACTCACTTATTTAAAATCCCTTTACCTGGATGGAAACCAGCTTCTAGAGATACCGCAGGGCCTTCCACCCAGCTTACAGCTTCTCAGCCTTGAGGCCAACAACATCTTTTCCATCAGAAAAGAGAATCTAACAGAACTGGCCAACATAGAAATACTCTATCTGGGCCAAAACTGTTATTATCGAAATCCTTGTTATGTTTCATATTCAATAGAAAAAGATGCCTTCCTAAACTTGACAAAGTTAAAAGTGCTCTCCCTGAAAGATAACAATGTCACAACCGTCCCTACTGTTTTGCCATCTACTTTAACAGAACTATATCTCTACAACAACATGATTGCAGAAATCCAAGAAGATGATTTTAATAACCTCAACCAATTACAAATTCTTGACCTAAGTGGAAATTGCCCTCGTTGTTATAATGCCCCATTTCCTTGTACGCCATGTAAAAATAATTCTCCCCTACAGATCCCTGTAAATGCTTTTGATGCGCTGACAGAATTAAAAGTTTTACGTCTACACAGTAACTCTCTTCAGCATGTGCCCCCAAGATGGTTTAAGAACATCAACAATCTCCAGGAACTAGATCTGTCCCAAAACTTCTTGGCCAAAGAAATTGGGGATGCCAAATTTCTGCATTTTCTCCCCAACCTCATCCAATTGGATCTGTCTTTCAATTTTGAACTTCAGGTCTATCGTGCATCTATGAATCTATCACAAGCATTTTCTTCACTGAAAAGCCTGAAAATTCTGCGGATCAGAGGATATGTCTTCAAAGAGCTGAAAAGCTTTAACCTCTCTCCATTACACAATCTTCAAAATCTTGAAGTTCTTGATCTTGGTACTAACTTTATAAAAATTGCTAACCTCAGCATGTTTAAACAATTTAAAAGATTGAAAGTCATAGATCTTTCAGTGAATAAAATATCACCTTCAGGAGATTCAAGTGAAGTTGGCTTCTGCTCAAATGCCAGAACTTCTGTAGAAAGTTATGAACCCCAGGTCCTGGAACAATTATATTATTTCAGATATGATAAGTATGCAAGGAGTTGCAGGTTCAAAAACAAAGAGGCTTCTTTCACGTCTGTTAATGAAAGCTGCTACAAGTATGGGCAGACCTTGGATTTAAGTAAAAATAGTATATTTTTTATCAAGTCCTCTGATTTT

>Hap_3

CCAGCGTCCTTTCACAGACTGGTCCATCTGGTAGAGATCGATTTCAGATGCAACTGTGTACCTATTCGATTGGGGTCAAAAAGCAACATGTGCCCCAGGAGGCTGCAGATTAAACCCAGAAGCTTTAGTGGACTCACTTATTTAAAATCCCTTTACCTGGATGGAAACCAGCTTCTAGAGATACCGCAGGGCCTTCCACCCAGCTTACAGCTCCTCAGCCTTGAGGCCAACAACATCTTTTCCATCAGAAAAGAGAATCTAACAGAACTGGCCAACATAGAAATACTCTATCTGGGCCAAAACTGTTATTATCGAAATCCTTGTTATGTTTCATATTCAATAGAAAAAGATGCCTTCCTAAACTTGACAAAGTTAAAAGTGCTCTCCCTGAAAGATAACAATGTCACAACCGTCCCTACTGTTTTGCCATCTACTTTAACAGAACTATATCTCTACAACAACATGATTGCAGAAATCCAAGAAGATGATTTTAATAACCTCAACCAATTACAAATTCTTGACCTAAGTGGAAATTGCCCTCGTTGTTATAATGCCCCATTTCCTTGTACGCCATGTAAAAATAATTCTCCCCTACAGATCCCTGTAAATGCTTTTGATGCGCTGACAGAATTAAAAGTTTTACGTCTACACAGTAACTCTCTTCAGCATGTGCCCCCAAGATGGTTTAAGAACATCAACAATCTCCAGGAACTAGATCTGTCCCAAAACTTCTTGGCCAAAGAAATTGGGGATGCCAAATTTCTGCATTTTCTCCCCAACCTCATCCAATTGGATCTGTCTTTCAATTTTGAACTTCAGGTCTATCGTGCATCTATGAATCTATCACAAGCATTTTCTTCACTGAAAAGCCTGAAAATTCTGCGGATCAGAGGATATGTCTTCAAAGAGCTGAAAAGCTTTAACCTCTCTCCATTACACAATCTTCAAAATCTTGAAGTTCTTGATCTTGGTACTAACTTTATAAAAATTGCTAACCTCAGCATGTTTAAACAATTTAAAAGATTGAAAGTCATAGATCTTTCAGTGAATAAAATATCACCTTCAGGAGATTCAAGTGAAGTTGGCTTCTGCTCAAATGCCAGAACTTCTGTAGAAAGTTATGAACCCCAGGTCCTGGAACAATTATATTATTTCAGATATGATAAGTATGCAAGGAGTTGCAGGTTCAAAAACAAAGAGGCTTTTTTCACGTCTGTTAATGAAAGCTGCTACAAGTATGGGCAGACCTTGGATCTAAGTAAAAATAGTATATTTTTTATCAAGTCCTCTGATTTT

>Hap_4

CCAGCGTCCTTTCACAGACTGGTCCATCTGGTAGAGATCGATTTCAGATGCAACTGTGTACCTATTCGATTGGGGTCAAAAAGCAACATGTGCCCCAGGAGGCTGCAGATTAAACCCAGAAGCTTTAGTGGACTCACTTATTTAAAATCCCTTTACCTGGATGGAAACCAGCTTCTAGAGATACCGCAGGGCCTTCCACCCAGCTTACAGCTCCTCAGCCTTGAGGCCAACAACATCTTTTCCATCAGAAAAGAGAATCTAACAGAACTGGCCAACATAGAAATACTCTATCTGGGCCAAAACTGTTATTATCGAAATCCTTGTTATGTTTCATATTCAATAGAAAAAGATGCCTTCCTAAACTTGACAAAGTTAAAAGTGCTCTCCCTGAAAGATAACAATGTCACAACCGTCCCTACTGTTTTGCCATCTACTTTAACAGAACTATATCTCTACAACAACATGATTGCAGAAATCCAAGAAGATGATTTTAATAACCTCAACCAATTACAAATTCTTGACCTAAGTGGAAATTGCCCTCGTTGTTATAATGCCCCATTTCCTTGTACGCCATGTAAAAATAATTCTCCCCTACAGATCCCTGTAAATGCTTTTGATGCGCTGACAGAATTAAAAGTTTTACGTCTACACAGTAACTCTCTTCAGCATGTGCCCCCAAGATGGTTTAAGAACATCAACAATCTCCAGGAACTAGATCTGTCCCAAAACTTCTTGGCCAAAGAAATTGGGGATGCCAAATTTCTGCATTTTCTCCCCAACCTCATCCAATTGGATCTGTCTTTCAATTTTGAACTTCAGGTCTATCGTGCATCTATGAATCTATCACAAGCATTTTCTTCACTGAAAAGCCTGAAAATTCTGCGGATCAGAGGATATGTCTTCAAAGAGCTGAAAAGCTTTAACCTCTCTCCATTACACAATCTTCAAAATCTTGAAGTTCTTGATCTTGGTACTAACTTTATAAAAATTGCTAACCTCAGCATGTTTAAACAATTTAAAAGATTGAAAGTCATAGATCTTTCAGTGAATAAAATATCACCTTCAGGAGATTCAAGTGAAGTTGGCTTCTGCTCAAATGCCAGAACTTCTGTAGAAAGTTATGAACCCCAGGTCCTGGAACAATTATATTATTTCAGATATGATAAGTATGCAAGGAGTTGCAGGTTCAAAAACAAAGAGGCTTCTTTCACGTCTGTTAATGAAAGCTGCTACAAGTATGGGCAGACCTTGGATCTAAGTAAAAATAGTATATTTTTTATCAAGTCCTCTGATTTT
